# Hydrogen-Bonded Complexes in Binary Mixture of Imidazolium-Based Ionic Liquids with Organic Solvents

**DOI:** 10.1021/acs.jpcb.3c05152

**Published:** 2023-10-09

**Authors:** Kaiyah Rush, Md Muhaiminul Islam, Sithara U. Nawagamuwage, Jorden Marzette, Olivia Browne, Kayla Foy, Khale’ Reyes, Melissa Hoang, Catherine Nguyen, Alexis Walker, Susana Ferrufino Amador, Emanuela Riglioni, Igor V. Rubtsov, Kevin Riley, Samrat Dutta

**Affiliations:** † Department of Chemistry, Xavier University of Louisiana, New Orleans, Louisiana 78125, United States; ‡ Department of Chemistry, Tulane University, New Orleans, Louisiana 70118, United States

## Abstract

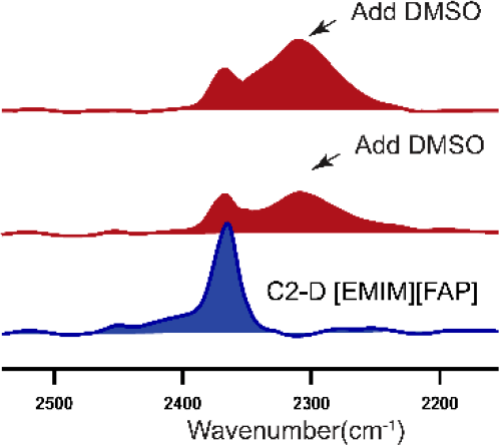

Though local structures in ionic liquids are dominated by strong Coulomb forces, directional hydrogen bonds can also influence the physicochemical properties of imidazolium-based ionic liquids. In particular, the C-2 position of the imidazolium cation is acidic and can bind with suitable hydrogen bond acceptor sites of molecular solvents dissolved in imidazolium-based ionic liquids. In this report, we identify hydrogen-bonded microenvironments of the model ionic liquid, 1-ethyl-3-methylimidazolium tris(pentafluoroethyl) trifluorophosphate, and the changes that occur when molecular solvents are dissolved in it by using a C–D infrared reporter at the C-2 position of the cation. Our linear and nonlinear infrared experiments, along with computational studies, indicate that the molecular solvent dimethyl sulfoxide can form strong hydrogen-bonded dimers with the cation of the ionic liquid at the C-2 position. In contrast, acetone, which is also a hydrogen bond acceptor similar to dimethyl sulfoxide, does not show evidence of cation–solvent hydrogen-bonded conformers at the C-2 position. The outcome of our study on a broad scale strengthens the importance of cation–solute interactions in ionic liquids.

## 1 Introduction

Binary mixtures of imidazolium-based ionic liquids with molecular solvents can give rise to attractive physicochemical properties that can open new applications for these liquids.^1^ However, predicting the ionic liquid’s molecular structure when mixed with a molecular solvent is not straightforward.^2−4^ Unlike molecular solvents, imidazolium-based ionic liquids are complex, spatially heterogeneous, non-aqueous liquids entirely made of ions.^5^ Mixing an ionic liquid with a molecular solvent causes electrostatic screening between the cation and anion, disrupting the ionic liquid’s local structures. Though nonspecific electrostatic interactions dominate in such mixtures, specific electrostatic interactions, such as hydrogen bonding, can arise between the ionic liquid and the molecular solvent that may lead to preferential solvation.^6^ We hypothesize that the characteristics of hydrogen-bonding interactions depend on the chemical identity of the molecular solvent. To prove the premise, we study the changes in the microenvironment of the model ionic liquid, 1-ethyl-3-methylimidazolium tris(pentafluoroethyl) trifluorophosphate ([EMIM][FAP]), when the molecular solvent, acetone or dimethyl sulfoxide (DMSO), is added. Both acetone and DMSO have hydrogen-bonding acceptor (HBA) sites and are thus capable of interacting with the acidic proton at the C-2 position of the imidazolium cation. Our experimental studies using the C–D infrared label at the C-2 position show evidence of the formation of cation–DMSO hydrogen-bonded species at the C-2 position but not with acetone, indicating the importance of preferential solvation in imidazolium-based ionic liquids.

The structure and dynamics of imidazolium-based ionic liquid mixtures depend on the concentration and the nature of the molecular solvent (note that “solvent” here refers to a molecular species in solution with an ionic liquid, whether it is the major or minor component).^7^ Binary mixtures of imidazolium-based ionic liquids with organic cosolvents can give rise to a multitude of structures, including, but not limited to, isolated molecules or aggregated forms of the ionic liquids, aggregated clusters or isolated forms of solvent molecules, hydrogen-bonded complexes between the ions of the imidazolium-based ionic liquid and the solvent, and other diverse, complex microstructures.^7−10^ In particular, competitive solvation of constituent ions of the ionic liquid by molecular solvents via hydrogen bonding is important and can dictate the physicochemical characteristics of these mixtures.^11,12^ Peculiar dissolution of cellulose in imidazolium-based ionic liquids mixed with DMSO or acetone highlights the significant role of imidazolium cation–molecular solvent hydrogen-bonding interactions on solution properties.^13,14^ Whereas DMSO added to an imidazolium-based ionic liquid dramatically accelerates the dissolution of cellulose, acetone on the other hand regenerates the dissolved cellulose. Thus, understanding the nature of cation–molecular solvent hydrogen bonding in binary mixtures is important not only from the perspective of cellulose dissolution but also for upcoming novel applications, such as their use as electrolytes in Li ion batteries.^15,16^ However, there is limited experimental information about the characteristics of these hydrogen-bonding microenvironments of ionic liquid–molecular solvent binary mixtures, especially in relation to the cation.

Imidazolium-based ionic liquids have aromatic hydrogen sites (C2–H, C4–H, and C5–H) on the cation, which can potentially bind with HBA sites of molecular solvents, but the strength of the hydrogen bonds depends on the electron-donicity of HBA sites.^17^ The C2–H site of the imidazolium cation of the ionic liquid is acidic^18^ and often is the preferred binding site of an HBA molecular solvent. In the structurally similar solvents DMSO and acetone, charge-enhanced C–H···O hydrogen bond interactions at the C2–H position of the imidazolium cation are expected.^19,20^ However, the strengths and nature of such hydrogen-bonded complexes in DMSO–ionic liquid and acetone–ionic liquid may differ in the same way, as observed in binary mixtures of DMSO and acetone with other molecular solvent systems. For example, it was shown that the time scales of the hydrogen bond dynamics of DMSO with chloroform are longer than those of acetone and chloroform.^21^ In other words, DMSO hydrogen bond interactions with the solute chloroform differ from those of acetone. In ionic liquids, such behavior may show concentration dependence.^22^ For example, there is evidence that at low concentrations of DMSO in ionic liquids, there is a strong solvation effect of DMSO in stabilizing a Brønsted acid. However, such trends collapse in larger fractions of DMSO in ionic liquids (high dilution regime).^23^ The suggested mechanism is the formation of cation–DMSO hydrogen-bonded complexes competing with cation–anion interactions at low concentrations of the molecular solvent. Marekha et al.^24^ suggested that hydrogen bonds at the C-2 position with the molecular solvent are likely to occur in ionic solvents at high dilutions. Seddon and Jitvisate^25^ reported on the preferential solvation of imidazolium cations by the molecular solvent DMSO, possibly via hydrogen bond interactions in dilute ionic liquid solutions. On the other hand, using molecular dynamic simulations, Zhao et al.^26^ concluded that there are no preferential interactions of the ionic liquid with DMSO. Instead, the ionic liquid in DMSO resembles solvent-surrounded ion pairs along with a small number of free ions. The presence of similar hydrogen-bonded structures is not ruled out for acetone–ionic liquids.^20^ At the same time, the study of Noack et al.^27^ on acetone–ionic liquid binary systems suggests that acetone can arrange itself on the top and the bottom of the imidazolium ring instead of forming hydrogen bonds with imidazolium protons. In other words, there are disparate views on hydrogen bonding in ionic liquid–molecular liquid binary systems.

**Figure 1:**
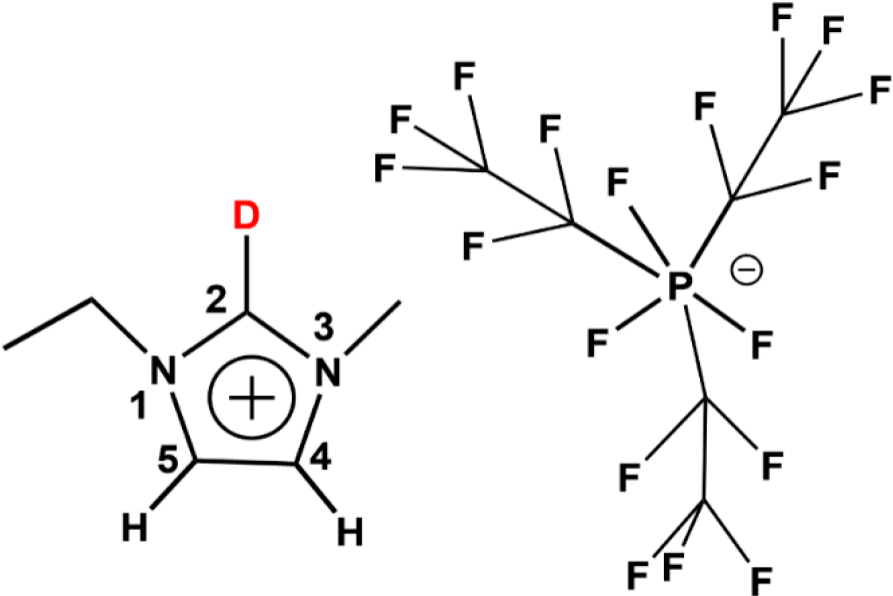
C2–D-labeled [EMIM][FAP]. The C-2 position is highlighted.

Isotope editing of the C2–H of imidazolium cations to C2–D provides a unique opportunity to observe changes in imidazolium ionic liquids at the C-2 position upon dilution with a molecular solvent by Fourier transform infrared spectroscopy (FTIR) from the perspective of the cation. Earlier, our group showed that this band is sensitive to the local microenvironment, particularly to hydrogen bond interactions, and temperature-induced reorganization.^28,29^ Moreover, the C–D stretching peak is amenable to nonlinear vibrational spectroscopy. In this study, we use the C–D peak at the C-2 position on the cation (Figure 1) of the model ionic liquid ([EMIM][FAP]) to investigate the changes that occur on adding a molecular solvent (either DMSO or acetone). We hypothesize that the formation and strength of intermolecular hydrogen bonding between the acidic C2–D of the cation of the ionic liquid and the HBA sites of DMSO and acetone will be different and can be assessed by the changes in the C–D infrared characteristics. Indeed, our infrared studies show that only DMSO forms hydrogen-bonded cation–solvent species, emphasizing the importance of the strength of the hydrogen bonging of the molecular solvent in solvating the ionic liquid. Further nonlinear infrared explorations clearly suggest the presence of three distinct hydrogen-bonded microenvironments in this DMSO–ionic liquid binary system.

## 2 Experimental Section

### 2.1 Reagents and Materials

The high-purity ionic liquids (99+%) D_2_O and NaOD used for the deuteration process were procured from Sigma-Aldrich, VWR, or Fischer Scientific. Anhydrous DMSO and acetone with <0.005% water were procured from the same sources.

### 2.2 Synthesis and Characterization of C2–D-Labeled [EMIM][FAP]

A detailed synthesis of the deuteration process is presented elsewhere.^30,31^ Briefly, the ionic liquid, [EMIM][FAP], was dissolved in D_2_O at 60 °C in a 1:30 molar ratio due to the poor solubility of this fluorinated compound in water. A catalytic amount (∼0.5 μM) of NaOD was added to this mixture. The solution was stirred under nitrogen for 24 h at 60 °C. The reaction was then cooled to about 4 °C in an ice bath. Cooling separated the deuterated ionic liquid from the bulk D_2_O, which was then carefully decanted into a clean vial. The product was dried under a high vacuum at 80 °C for at least 24 h, resulting in >90% yield. The conversion was estimated from the disappearance of the C2–H proton peak in its NMR spectrum, which was measured with a Bruker 300 MHz NMR instrument, as described in our earlier reports (see Figure S1). The water content in the samples, determined through the “free water” bands at 3640 and 3560 cm^–1^ both for pure ionic liquids and mixtures, was in the micromolar range and was found using the same procedure as reported earlier.^28,29^

### 2.3 FTIR Studies of C2–D-Labeled [EMIM][FAP] with Solvents

The molecular solvents were dried before mixing with the C2–D-labeled liquid. Solvent mixtures were prepared by mixing the ionic liquid with molecular solvents at different volumetric ratios. Every ratio of the mixed liquid was tested with an FTIR spectrometer (Bruker, Tensor II) at least thrice. In a typical experiment, approximately 10 μL of the ionic liquid mixture was placed between two CaF_2_ windows separated by a 25 μm spacer and sealed in a gas-tight dismountable liquid cell holder (Pike Technologies). FTIR spectra were collected over 16 scans with a resolution of 2 cm^–1^. Analysis of the spectra was done with the OPUS software.

### 2.4 Two-Dimensional Infrared Spectroscopy

An in-house-built, fully automated, dual-frequency, three-pulse photon echo two-dimensional infrared (2DIR) spectrometer with heterodyned detection was used to obtain absorptive 2DIR spectra. Details of the instrument are described elsewhere.^32^ In short, laser pulses of 800 nm wavelengths with a 1 kHz repetition rate (Libra, Coherent) were used to pump two OPAs (optical parametric amplifiers) (Palitra, Quantronix). Each OPA is followed by a DFG (difference frequency generation) unit, producing vertically polarized mid-IR pulses with an ∼1 μJ pulse energy. Each mid-IR beam was split into two parts, forming three beams interacting with the sample and a local oscillator. The instrument uses an automatic beam direction stabilization scheme accurate to 50 μrad.^33^ The three beams centered at ca. 2350 cm^–1^ were focused into the sample cell with a spot size of ∼100 μm in diameter.

Real-part rephasing and nonrephasing 2DIR spectra of the C–D stretching mode were measured, phased individually based on the pump–probe spectra of the respective transition and added, resulting in absorptive 2DIR spectra. The absorptive spectra were measured at different waiting times, *T*, which were the delays between the second and third mid-IR pulses interacting with the sample. A central line slope (CLS) of the 0 → 1 transition was determined for the absorptive peaks for each waiting time. The measurements were performed at room temperature, 22.5 ± 0.5 °C, in a sample cell with two 1 mm thick CaF_2_ windows and a 50 μm Teflon spacer.

### 2.5 Computational Methods

Electrostatic potential calculations for EMIM, acetone, and DMSO were computed on the 0.001 au contour of the molecular electronic density using Spartan at the BLYP/6-311G** level of theory, incorporating the PCM method with the option “nonpolar solvent”. All geometry optimizations, interaction energies, and harmonic frequency calculations are carried out using the B97M-V functional along with the may-cc-pVTZ basis set. The CPCM implicit solvation model is used for all computations to mimic the ionic liquid environment. The calculations described above were done using the ORCA suite of molecular electronic structure programs using tight SCF convergence (TIGHTSCF), tight geometry optimization (TIGHTOPT), and the DEFGRID3 numerical integration grid.

## 3 Results and Discussion

C2–D-labeled [EMIM][FAP] was synthesized in a straightforward way with over 90% conversion. The conversion was estimated from the disappearance of the C2–H proton peak in its NMR spectrum, as described in our earlier reports (see Figure S1).^28,29^ The water content determined by infrared spectroscopy, both for the pure solution and the mixture, was in the micromolar range.

**Figure 2:**
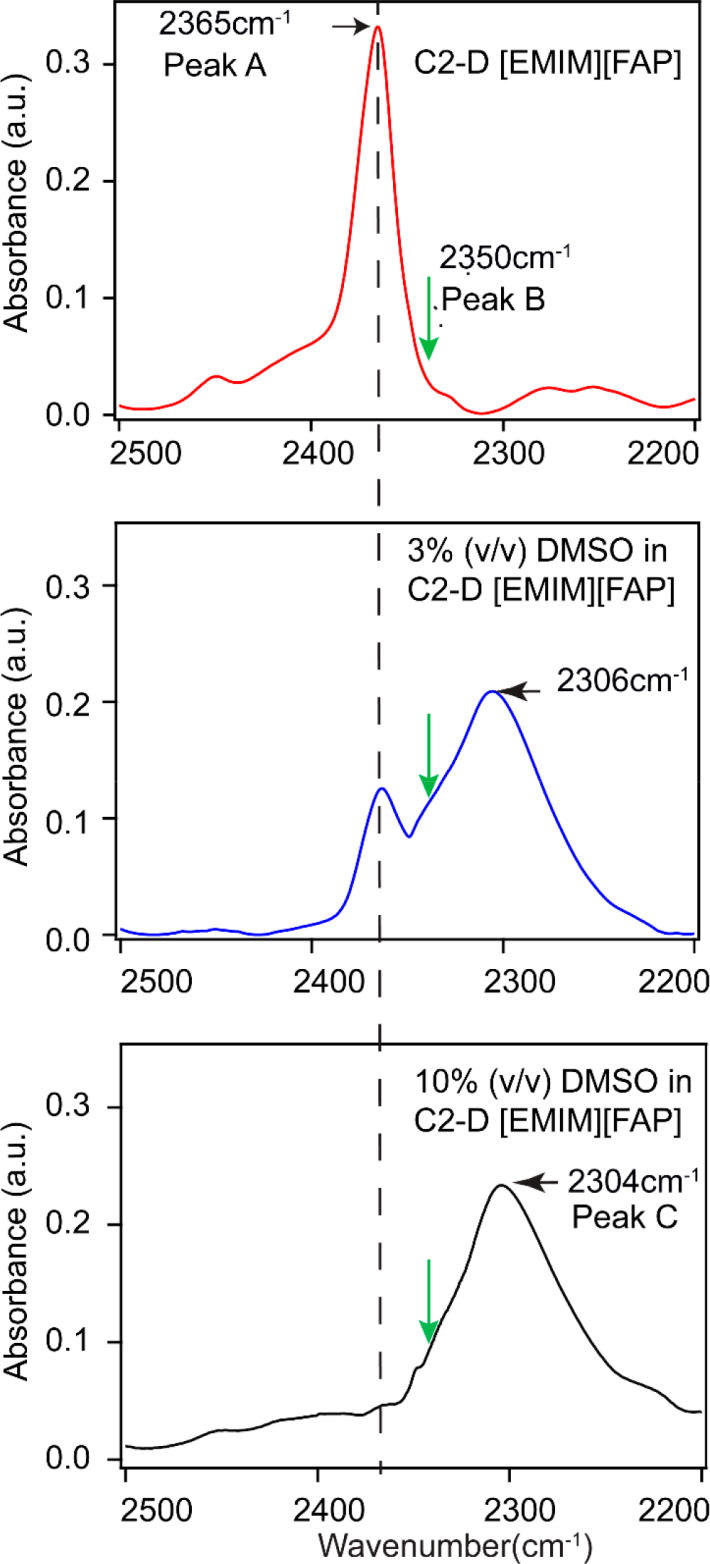
C–D band of [EMIM][FAP] at 2365 cm^–1^ (top), which splits upon adding DMSO (middle) and red-shifts by >60 cm^–1^ (bottom) to a single peak. The dilution percentage is approximate. The green arrow shows the approximate location of the hidden peak B, which was later revealed by 2DIR spectroscopy.

The FTIR spectrum of C2–D-labeled [EMIM][FAP] shows a peak at 2365 cm^–1^, denoted as peak A (Figure 2, top), featuring a narrow width of 16 cm^–1^, which is in line with our earlier studies.^28,29^ Beside the main C–D peak, there is another hidden transition, peak B (Figure 2, green arrow), which was later uncovered by 2DIR measurements. On adding DMSO, a red-shifted broad peak arises, centered at 2306 cm^–1^ and denoted as peak C (Figure 2 middle, bottom). The full width at half-maximum (fwhm) of this peak is ∼45 cm^–1^ (Figure 2, middle), as revealed by fitting with a Gaussian profile. The characteristics of peak A, the peak position, and the width remain the same in the presence of DMSO, albeit the amplitude is lower (see Figure S2). As the concentration of DMSO increases, the amplitude of peak A decreases and that of peak C increases (see Figure S3). At approximately 10% (v/v) DMSO, peak A completely disappears, and the spectrum becomes dominated by peak C (Figure 2, bottom). Note that at 10% (v/v) of DMSO, the molar ratio of DMSO molecules to the ionic liquid is greater than 1. We see a similar trend in C2–D-labeled 1-ethyl-3-methylimidazolium bis(trifluoromethylsulfonyl)imide ([EMIM][Tf_2_N]), but the frequency separation of the second peak, appearing upon DMSO addition, is not as large as for the C2–D-labeled [EMIM][FAP] (see Figure S4). Interestingly, upon the addition of acetone to the C2–D-labeled [EMIM][FAP], we do not see a rise of the separate, red-shifted peak, even when the acetone amount exceeds 10% (v/v) (see Figure S5). The C–D peak positions of the neat ionic liquid and the acetone-added ionic liquid are identical at 2365 cm^–1^, even when acetone is in excess. However, the peak becomes broader in the mixture featuring a lower amplitude. The results show that the similar molecular solvents, DMSO and acetone, interact differently at the C-2 position in [EMIM][FAP].

**Figure 3:**
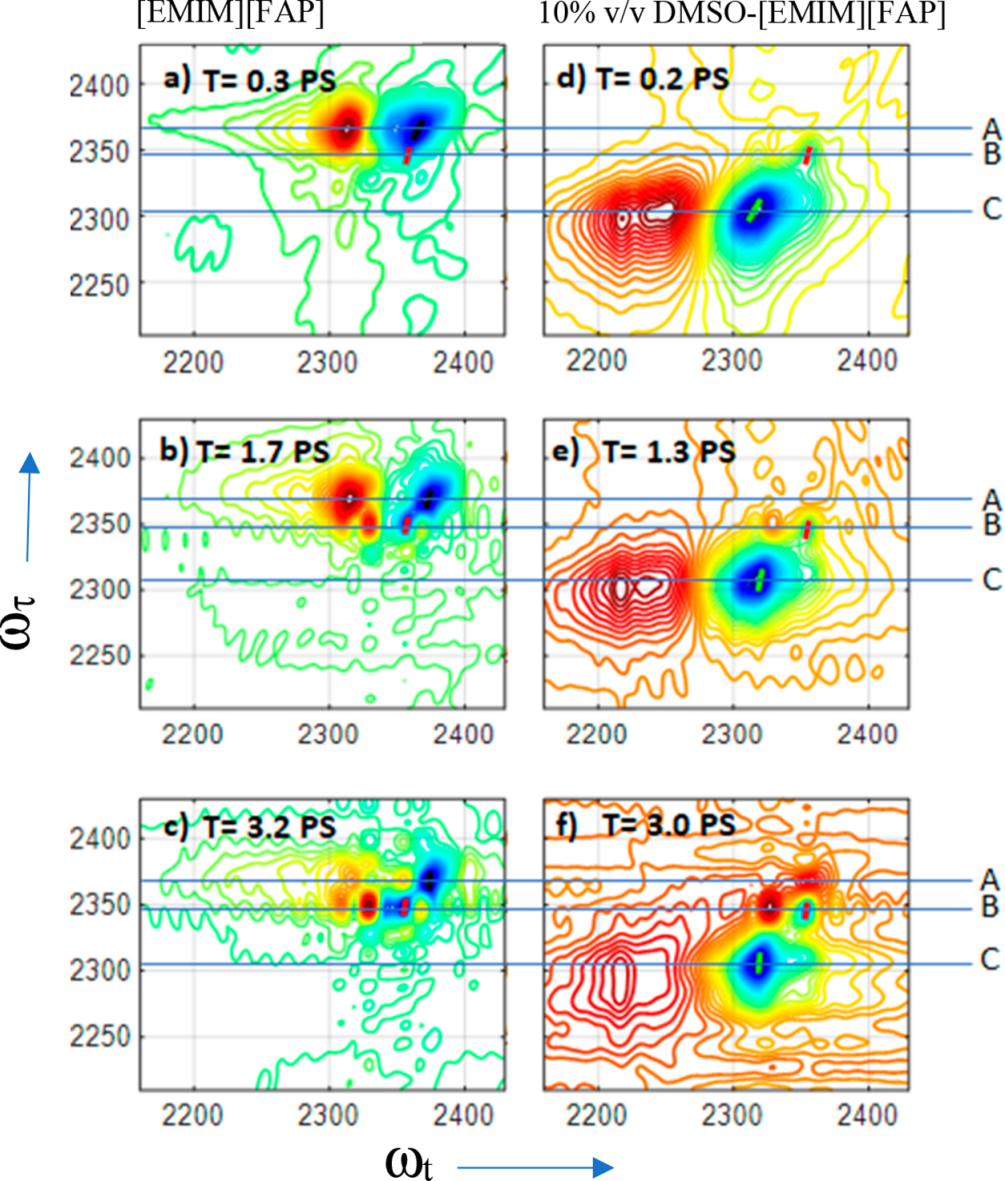
Absorptive 2DIR spectra of C2–D-labeled pure [EMIM][FAP] (a–c) and C2–D-labeled [EMIM][FAP] with 10% v/v DMSO (d–f). The guides A, B, and C represent peak A (2365 cm^–1^), peak B (2350 cm^–1^), and peak C (2304 cm^–1^). The representative lines (black, red, and green) on the diagonal peaks from which the centerline slopes were calculated are also shown for each 2DIR snapshot.

To further investigate the interactions between DMSO and C2–D-labeled [EMIM][FAP], we performed 2DIR measurements in the diagonal region of the C–D stretching mode. Representative 2DIR spectra of the neat ionic liquid and its mixture with DMSO (10% (v/v)) are shown in Figure 3. These 2DIR snapshots have several general characteristics. The negative diagonal peaks (blue) represent the ground-state bleach (0 → 1) and stimulated emission (1 → 0) contributions, and the positive peaks (red) represent excited-state absorption (ESA, 1 → 2). The ESA peaks appear at lower frequencies due to the anharmonicity (Δ) of the vibrational transitions.

The 2DIR spectrum of the neat ionic liquid at small waiting times is dominated by the broad features of peak A showing the negative peak centered at ω_*t*_ = 2365 cm^–1^ and the positive peak at ω_*t*_ = 2313 cm^–1^ (Figure 3a). An additional narrow peak, centered at ca. 2350 cm^–1^, denoted as B, is apparent but not fully resolved at small waiting times. At longer waiting times, peak B becomes well-resolved (Figures 3 b and 3c) for neat C2–D-labeled [EMIM][FAP]. The appearance in the 2DIR spectra of peak B, which is not obvious in the linear spectrum (Figure 2, green arrow), highlights the advantage of 2DIR to show hidden features. The anharmonicity values for peaks A and B are 52 ± 1 and 26 ± 1 cm^–1^, respectively. The 2DIR spectra of the sample with 10% v/v DMSO are dominated by the peak C (2304 cm^–1^) contributions (Figures 3d–3f). The anharmonicity of peak C is found at ca. 85 ± 10 cm^–1^, which is significantly larger than those of peak A and peak B in neat C2–D-labeled [EMIM][FAP]. In addition, peak B appears in the 2DIR spectra of the sample with DMSO, similar to that for neat ionic liquids. Interestingly, the characteristics of peak B do not change, including its central frequency and anharmonicity. The lifetimes of peaks A, B, and C, determined by integrating their ESA peaks and plotting the result as a function of *T*, are 1.0 ± 0.1, 1.3 ± 0.1, and 1.2 ± 0.1 ps, respectively. Note that the 2DIR spectra of mixtures of intermediate concentrations of the ionic liquid with DMSO show the presence of all three peaks.

**Figure 4:**
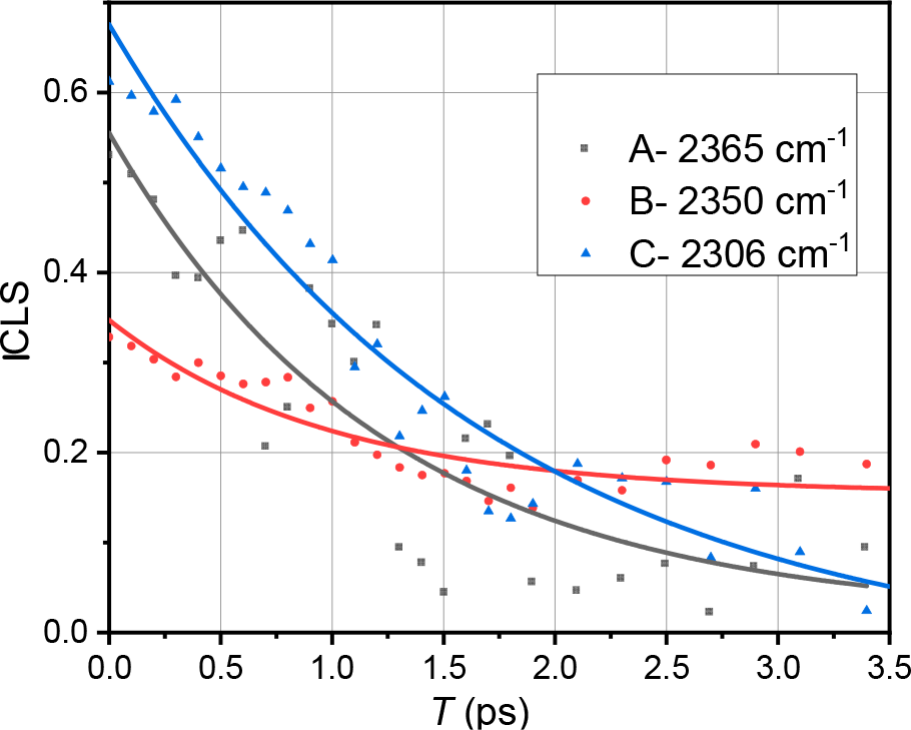
ICLS decays for the C–D stretching mode of deuterated C-2 for peaks A (black) and B (red), which were determined for [EMIM][FAP], and peak C (blue), which was determined for 10% (v/v) DMSO. Note that the peak B spectral diffusion was also determined from the10% (v/v) DMSO sample, and the data were similar within the noise level.

To characterize the environments that resulted in different C–D peaks in the samples, we analyzed the spectral diffusion for peaks A and B from C2–D-labeled pure [EMIM][FAP] and peak C from 10% v/v DMSO. Spectral diffusion provides the time-dependent fluctuations of the C–D vibration due to local environment dynamics. At a short waiting time *T,* the diagonal peaks A, B, and C are diagonally elongated because of the inhomogeneity of the transitions. At later waiting times, the correlation between the frequencies of the excited and probed transitions is lost to frequency fluctuations, resulting in a rounder peak. Typically, the frequency–frequency correlation function (FFCF) is used to describe spectral diffusion dynamics.^34^ In this work, we use the inverse center line slope (ICLS) of the investigated peaks to represent the normalized inhomogeneous contribution to the FFCF.^35^ The waiting time dependence of the ICLS for each peak shown in Figure 4 was fitted with the single exponential function: ICLS(*T*) **=***A*e^(−*T*/τ)^*+ y*_0_ (Figure 4). The obtained fit parameters are shown in Table 1. As seen in Figure 4, peaks A and C have a higher initial ICLS value compared with peak B, with their traces completely decaying to zero within 3.5 ps. In contrast, the ICLS of peak B decays to only 40% of the initial value within our experimental time window. Note that the data become increasingly noisy at longer waiting times due to the short lifetime of the C–D stretching mode, so 2DIR data are not presented for time delays exceeding 3.5 ps.

**Table 1:** Table of FFCF Parameters

frequency (cm^-1^)	*A* + *y*_0_	*y*_0_	τ (ps)
A-2365	0.54 ± 0.06	±0.07	1.2 ± 0.4
B-2350	0.35 ± 0.02	±0.01	1.0 ± 0.2
C-2306	0.71 ± 0.05	0.03 ± 0.06	1.6 ± 0.3

Hydrogen bond interactions between imidazolium cations and their paired anions play a role in the structure and dynamics of ionic liquids. In our earlier work,^28^ we showed through experiments and simulations that hydrogen bonding between imidazolium cations at the C-2 position and suitable hydrogen bond acceptor sites on anions influences the C–D infrared frequency of C2–D-labeled imidazolium-based ionic liquids. DFT calculations by others^36,37^ on [EMIM][FAP] show that hydrogen bond interactions exist between the cation and alkyl fluorine atoms of the anion and are the strongest at the C-2 hydrogen of the cation. A recent review from Fayer’s group^38^ suggests that in typical ionic liquids, there is a subtle balance between a small number of stronger, directional hydrogen bond interactions between the cations and anions, leading to shorter H-bonds, and a large number of weaker hydrogen bond interactions between them, leading to looser H-bonds. All this information, coupled with the experimental observations, enables us to propose molecular-level structures of the investigated systems. We assign peak A (∼2365 cm^–1^) to weak hydrogen-bonded species, whereas peak B (∼2350 cm^–1^) is assigned to strongly hydrogen-bonded cation and anion conformers in neat [EMIM][FAP]. Our hypothesis is that peak A originates from weak hydrogen bonding at the C-2 position of the cation with the fluorine atoms of the alkyl [FAP] side chain. The addition of DMSO results in the competitive disappearance of peak A and the appearance of peak C at ∼2304 cm^–1^. This observation allows us to conclude that weak cation–anion hydrogen bond interactions in neat [EMIM][FAP] are replaced by stronger hydrogen-bonded cation–DMSO interactions, resulting in peak C. The large red-shift of the C–D frequency from peak A to peak C observed in the experiments supports the conclusion that the cation–DMSO interaction is stronger. In addition, the C–D shift(s) of the [EMIM]···DMSO hydrogen-bonded dimer from the [EMIM] base frequency, calculated using computational methods (discussed later), approximately matches our experimentally observed red-shifts, reaffirming the formation of strong hydrogen-bonded cation–DMSO conformers. Further support for our molecular picture of peaks A and C comes from their large difference anharmonicity values, which were ∼52 and ∼85 cm^–1^, respectively. Substantial work from Sandorfy’s^39,40^ group shows that anharmonicity increases with an increase in the hydrogen bond strength. Such information strengthens the idea that peak A represents weak hydrogen-bonded cation–anion species, whereas peak C represents a strong, hydrogen-bonded, and solvent-separated cation–DMSO ion pair system. Peak B, as mentioned earlier, represents strong cation–anion hydrogen-bonded species. The observation that the addition of DMSO does not change the peak B characteristics supports this assignment. However, conformers associated with peak B are different than conventional hydrogen-bonded structures, as the anharmonicity associated with it is smaller (∼26 cm^–1^) than either peak A or peak C. Tokmakoff and colleagues^41^ have recently shown that under certain conditions, a decrease in anharmonicity can be an indication of strong hydrogen bonding. A possible structure that can represent peak B involves the imidazolium cation forming strong hydrogen bonds with fluorine atoms bonded to the phosphorus atom in the [FAP] anion. The fluorine atoms bonded to the phosphorus atom are significantly more electronegative than the fluorine atoms of the alkyl side chain and thus can form strong cation–anion hydrogen-bonded conformers. In summary, we identified three distinct hydrogen-bonded microenvironments in the DMSO–ionic liquid binary mixtures.

The FFCF analysis provides additional information about the C–D bond dynamics at the C-2 position in the binary mixtures (Figure 4). The FFCFs for peaks A and C show large inhomogeneous broadening (Table 1). The observations reinforce the idea that the peaks represent hydrogen-bonded conformers, as hydrogen bonding is typically associated with strong inhomogeneous broadening.^42^ Peak C, assigned to the DMSO–cation complex, has the largest inhomogeneity, which is indicative of overall greater heterogeneity. Recall that peak C at ∼2306 cm^–1^ (fwhm ≈ 50 cm^–1^) is broader than peak A at ∼2365 cm^–1^ (fwhm ≈ 16 cm^–1^). This line width broadening associated with peak C is consistent with greater heterogeneity in the environment experienced by a C–D probe in the DMSO–cation complex in our 2DIR experiments. The spectral diffusion times for peak A (1.2 ± 0.4 ps) and peak C (1.6 ± 0.3 ps) are similar and in line with hydrogen bond dynamics reported in other systems.^43,44^ Unlike peaks A and C, the cation–anion hydrogen-bonded conformers represented by peak B at ∼2350 cm^–1^ are unique and show lower peak inhomogeneity (Figure 4). Moreover, the inhomogeneous ensemble of oscillators, which generates peak B, does not fully randomize within 3.5 ps. The ICLS of peak B decays only to 40% of the initial value within this time window. In contrast, the FFCFs of peaks A and C decay to zero within the same time period. The slow decay of the FFCF supports the assumption that peak B represents a small subset of strong hydrogen-bonded cation–anion conformers in ionic liquids.^38^ In conclusion, the anharmonicities, inhomogeneity, and spectral diffusion dynamics measured by 2DIR spectroscopy reveal the differences in the heterogeneity and motion^45^ of the involved hydrogen-bonded species, indicating the extent of heterogeneity in DMSO–ionic liquid binary mixtures. Such heterogeneity was earlier reported in the literature for binary mixtures of DMSO with molecular solvents.^46^ Our analysis of the C–D dynamics helps characterize different conformers in the investigated systems.

Considering that peaks A–C represent different hydrogen-bonding microenvironments of DMSO–[EMIM][FAP] at the C-2 position of the cation, it is reasonable to assume that there can be chemical exchange among these microenvironments. Such exchanges, if present, should result in an increase in the off-diagonal peaks in the cross-peak positions in the 2DIR spectra at later waiting times.^47,48^ An indication of such cross-peaks between peaks A and B and peaks C and B can be found in Figures 3c and 3f, respectively. For example, cross-peaks at a (ω_τ_, ω_*t*_) of (2365 cm^–1^, 2350 cm^–1^) and (2350 cm^–1^, 2365 cm^–1^) are developing in the 2DIR spectra of the pure ionic liquid at *T* = 3.2 ps (Figure 3c). Similarly, a peak at (2306 cm^–1^, 2355 cm^–1^) appears in the 2DIR spectrum at *T* = 3 ps for the sample with DMSO. However, the time dependence of those chemical exchanges could not be extracted due to the overlapping of the peaks, and the main peak dominates.

**Figure 5:**
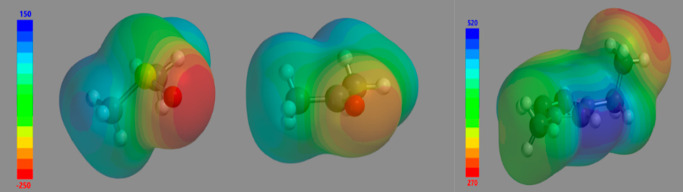
Electrostatic potential maps (kJ/mol) of DMSO (left), acetone (middle), and EMIM (right) computed at the BLYP/6-311G** level of theory. Note that the scale for EMIM is entirely positive.

The experimental evidence presented above suggests the presence of multiple hydrogen-bonded species in the [EMIM][FAP]–DMSO system, but the [EMIM][FAP]–acetone system is different. The C2–D peak position of [EMIM] in [EMIM][FAP] does not shift even when acetone is in excess, unlike the [EMIM][FAP]–DMSO system. To visualize the molecular picture of these ionic liquid mixtures in the context of the infrared results, we study the electrostatic potentials for [EMIM], DMSO, and acetone using the BLYP/6-311G** level of theory. As seen in Figure 5, the area of negative potential in the DMSO carbonyl group is substantially larger and more negative than that of the acetone carbonyl group, indicating that DMSO should serve as a better hydrogen bond acceptor. It is also seen that despite its overall positive charge, [EMIM] has a very anisotropic charge distribution, with C2–H being significantly more positive than other regions of the molecular ion. We therefore anticipate that the strongest hydrogen bonds occurring between [EMIM] and the two hydrogen bond acceptors (acetone and DMSO) will involve the C2–H hydrogen. In our earlier computational work,^28^ we showed that the C2–D peak position of the cation of imidazolium-based ionic liquid is sensitive to hydrogen bonding. The absence of a hydrogen bond interaction signature in the C2–D infrared band of the [EMIM]–acetone system suggests that other geometrical arrangements play a more dominant role.

**Figure 6:**
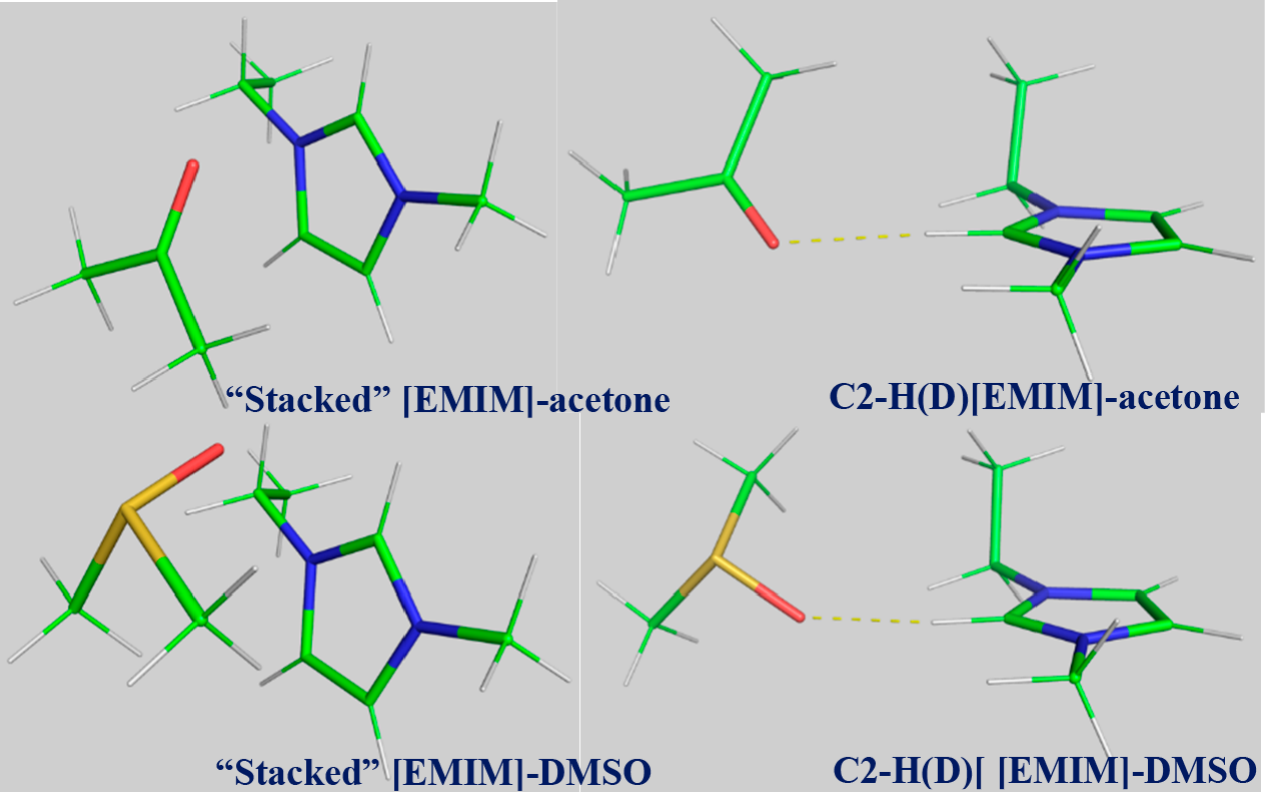
Optimized structures (ε = 12.5) of “stacked” and C2–H(D) hydrogen-bonding [EMIM]–acetone (top) and [EMIM]–DMSO (bottom).

In order to investigate the geometric preferences for binding of DMSO and acetone to [EMIM], we performed B97M-V/may-cc-pVTZ optimizations of the [EMIM]–DMSO and [EMIM]–acetone dimers starting from several starting geometries (Section S6). All calculations were run using the CPCM implicit solvation method to mimic the liquid environment. A challenge to running the calculations was the estimation of reasonable dielectric constant(s) of the ionic liquid–solvent mixtures. Recent studies show that the dielectric constant can vary as a function of molecular solvent concentration in the ionic liquid.^49^ As such, we conducted optimizations and interaction energy calculations of different starting geometries using six different dielectric constants ranging from 4.9 (chloroform) to 24.3 (ethanol). The starting geometries were chosen to represent several different binding motifs that are of particular interest in this study, namely, the ones involving the ring protons on [EMIM] and “stacked” interactions, in which acetone or DMSO is located above and below the imidazolium ring (here, “above” denotes a position on the same side as the ethyl tail). Examples of optimized structures are presented in Figure 6. Other data, including but not limited to interaction energies, vibrational shifts, and hydrogen bond energies, are given in Section S6.

**Figure 7:**
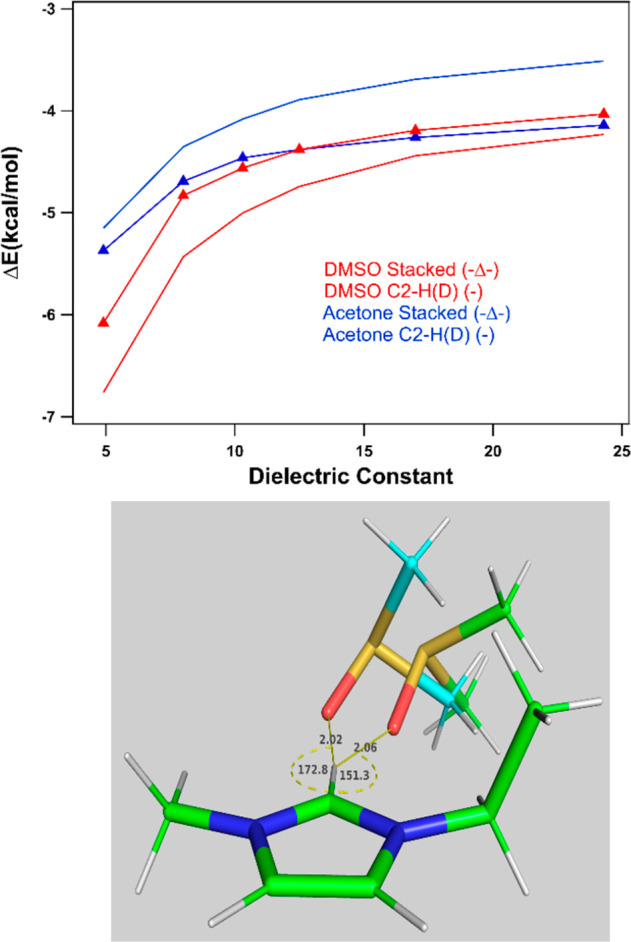
(top) Optimum interaction energies, as a function of the dielectric constant, for the hydrogen-bonded (C2–H(D)) and “stacked” [EMIM]–acetone and [EMIM]–DMSO structures. (bottom) Two configurations of the C2–H(D) hydrogen-bonding structure of [EMIM]–DMSO (ε = 12.5). (1) Optimal interaction energy structure (green carbons). (2) Structure corresponding to the maximum infrared red-shift (blue carbons). Distances are in Angstroms, and angles are in degrees.

The results presented in Figure 7 (top) show that the interaction strength of the dimers is a relatively strong function of the dielectric constant, with the magnitude of the interaction energy decreasing with increasing the dielectric constant. As seen in Figure 7 (top), the “stacked” configuration of the [EMIM]–acetone complex is favored over the hydrogen-bonded configuration. The “stacked” structures, whether it is with DMSO or acetone, have a small impact on the vibrational shift at the C-2 position (see Section S6). The preference of the “stacked” [EMIM]–acetone complex over the hydrogen-bonded complexes with imidazolium ring protons was also noted by Noack and co-workers.^27^ On the other hand, the hydrogen-bonded configuration of the [EMIM]–DMSO complex is favored over the “stacked” configuration. Our studies show that DMSO prefers the C-2 proton for hydrogen bonding when compared to other ring protons in the [EMIM]–DMSO complex, resulting in a large red-shift of the C2–D peak, which aligns with our experimental results. It should be noted that computed structures where acetone binds at the C-2 position of the cation show a similar red-shift as is observed for DMSO, but, as mentioned earlier, the “stacked” configuration is preferred by acetone and thus has limited influence on C2–D vibration. Another noteworthy feature of the C2–D hydrogen-bonding interaction of the [EMIM]–acetone or [EMIM]–DMSO complex is that the strongest hydrogen bonds are not associated with the largest C2–D red-shifts (see Section S6). Figure 7 (bottom) exemplifies this surprising finding. Here, the minimum energy geometry of the [EMIM]–DMSO complex (ε = 12.5) exhibits a relatively nonlinear C–H···O angle of 151.3°. The maximum vibrational red-shift geometry, on the other hand, has a much more linear hydrogen-bonding configuration, with a C–H···O angle of 172.8° and a slightly shorter H···O distance (2.02 Å compared with 2.06 Å). The possible reason for this somewhat unexpected result, where the preferred geometric configuration of the complex between the cation and the neutral molecule does not necessarily correspond directly to the most optimal hydrogen-bonding arrangement, may arise due to factors such as the alignment of the DMSO methyl and EMIM ethyl tails. In short, our computational assessment suggests that DMSO prefers hydrogen-bonded complexes with the ring protons, with the strongest bond at the C-2 position in [EMIM][FAP], whereas acetone prefers stacked structures with the cation of the ionic liquid.

In summary, our study of a DMSO–[EMIM][FAP] mixture with linear and nonlinear infrared spectroscopy shows evidence of several hydrogen-bonded microenvironments at the C-2 position of the cation, reaffirming the suitability of vibrational bands of ring protons for analyzing the structure and dynamics of ionic liquids.^50,51^ Many authors^24,25^ have speculated that DMSO–cation hydrogen-bonded species exist under a high dilution regime. Our studies show that such complexes exist in both high- and low-dilution regions. Computational studies in this work suggest that DMSO has a tendency to form hydrogen-bonded conformers at the C-2 position of the cation. The calculated red-shift(s) of the C–D peak at different dielectric constants are in line with the experimental observations. In acetone, we do not see a red-shift of the C–D vibration of the cation even at high dilution, suggesting that acetone is not forming hydrogen-bonded species at the C-2 position of the cation. Our computational studies suggest that acetone prefers to form “stacked” structures, where acetone is arranged at the top and the bottom of the imidazolium ring, which is in line with the report by Noack et al.^27^ Taken together, our exploration reveals several preferential solvation motifs of molecular solvents in imidazolium-based ionic liquids.

## Conclusion

Ionic liquids are complex solvents. Besides Coulombic forces, mixtures of molecular solvents with imidazolium-based ionic liquids can be influenced by hydrogen-bonding between the ionic liquid and the molecular solvent. With our model C2–D-labeled [EMIM][FAP] system, we observe a large red-shift of the C–D stretching peak at the C-2 position of the cation when DMSO is added to the ionic liquid compared to the C–D band of neat [EMIM][FAP], indicating the formation of a hydrogen-bonded [EMIM]–DMSO species. Two-dimensional IR analysis of the [EMIM][FAP]–DMSO system revealed three subsets of conformers with different spectral widths, diagonal anharmonicities, and spectral diffusion dynamics, demonstrating the presence of both strong and weak hydrogen-bonded species. Our analysis shows that the weaker hydrogen-bonded conformers involving the cation and the anion of the ionic liquid are replaced by stronger cation–DMSO hydrogen-bonded dimers, generating solvent-separated ions in the ionic liquid. The formation of strong cation–DMSO hydrogen-bonded complexes is supported by our computational studies. Interestingly, we also observe the presence of preserved hydrogen-bonded cation–anion conformers from 2DIR experiments in pure [EMIM][FAP] and the [EMIM][FAP]–DMSO mixture, with a small diagonal anharmonicity of ∼26 cm^–1^ and slow overall spectral diffusion dynamics. These subsets of ionic liquid conformers are resistant to changes associated with the addition of a molecular solvent such as DMSO. In contrast, the center C–D infrared peak of C2–D-labeled [EMIM][FAP] does not show a red-shift when acetone is added to the liquid, even at high dilutions. Computational studies reveal the formation of “stacked” structures between acetone molecule(s) and the cation. Taken together, our results suggest that even if similar hydrogen bond acceptor solutes (in our case DMSO and acetone) are dissolved in imidazolium-based ionic liquid, they may be solvated differently, showing the existence of solute-specific solvation in ionic liquids. Further studies are underway to explore whether aromatic carbonyl and sulfonyl groups interact differently from their alkane counterparts.

## Supporting Information Available

NMR data of ionic liquids, infrared profiles and Gaussian fits with different amounts of DMSO and deuterated [EMIM][FAP], additional infrared spectrum of [EMIM][Tf_2_N] in the presence of DMSO, and results from computational calculations for the investigated ionic liquid with DMSO and acetone (PDF)
