# Supplementary material for: Hydrogen-Bonded Complexes in Binary Mixture of Imidazolium-Based Ionic Liquids with Organic Solvents
Source: J Phys Chem B. 2023 Oct 9;127(41):8916–25. doi: 10.1021/acs.jpcb.3c05152 (PMC10591470; doi:10.1021/acs.jpcb.3c05152)
Supplement: Supplementary file 1 — jp3c05152_si_001.pdf [file jp3c05152_si_001.pdf]

Hydrogen-Bonded Complexes In Binary Mixture of Imidazolium-Based Ionic Liquids With Organic Solvents

Kaiyah Rush†, Md Muhaiminul Islam ‡*, Sithara U. Nawagamuwage‡, Jorden Marzette†, Olivia Browne†, Kayla Foy†, Khale' Reyes†, Melissa Hoang†, Catherine Nguyen†, Alexis Walker†, Susana Ferrufino Amador†, Emanuela Riglioni †, Igor V. Rubtsov‡, Kevin Riley †, and Samrat Dutta †#*

*Equal contributors

†Department of Chemistry, Xavier University of Louisiana, New Orleans, Louisiana, USA.
‡ Department of Chemistry, Tulane University, New Orleans, Louisiana, USA.

#email: sdutta@xula.edu

Phone: +1 504 520 5820

Table of Contents

S1. NMR of ring protons of native [EMIM][FAP] and C2-D labeled [EMIM][FAP].
S2. Gaussian fits of 3 % (v/v) DMSO and 10 % (v/v) in DMSO in C2-D labeled [EMIM][FAP].
S3. Infrared profile of C-D region of C2-D labeled [EMIM][FAP] on adding DMSO.
S4. Changes in C2-D lineshape of C2-D labeled [EMIM][Tf_2N] with DMSO.
S5. Changes of C-D lineshape of C2-D labeled [EMIM][FAP] with acetone.
S6. Computational results.

S1. NMR of ring protons of native [EMIM][FAP] and C2-D labeled [EMIM][FAP].

C2-H of imidazolium-based ionic liquids has a characteristic NMR singlet between 9-10 ppm. Similarly, C4-H and C5-H have two well defined peaks between 7-8 ppm. On conversion of C2-H to C2-D, the C2-H NMR signal decreases (or almost disappears). However, C4-H and C5-H do not change. Integration of C2-H peak area with methyl group in the imidazolium moiety shows that the conversion is >90% to C2-D product.

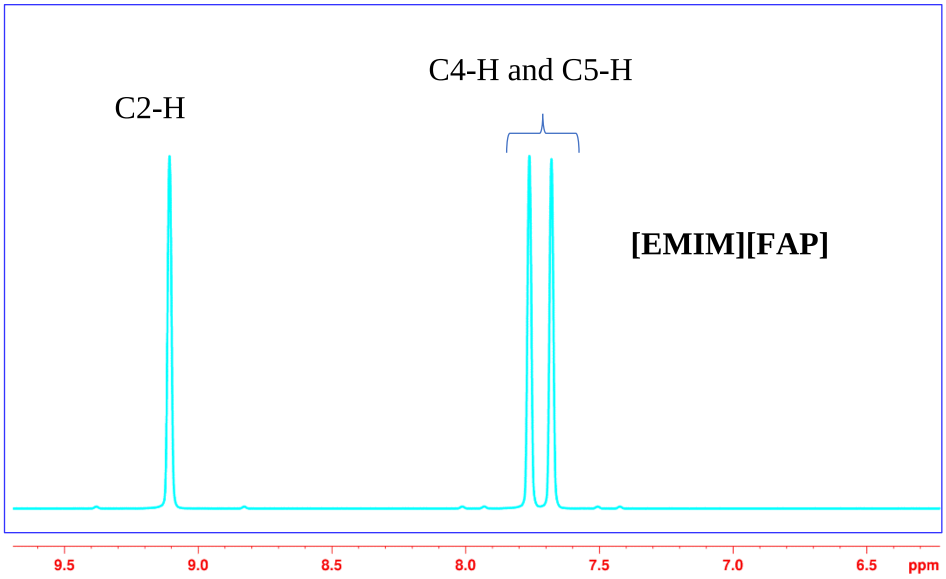

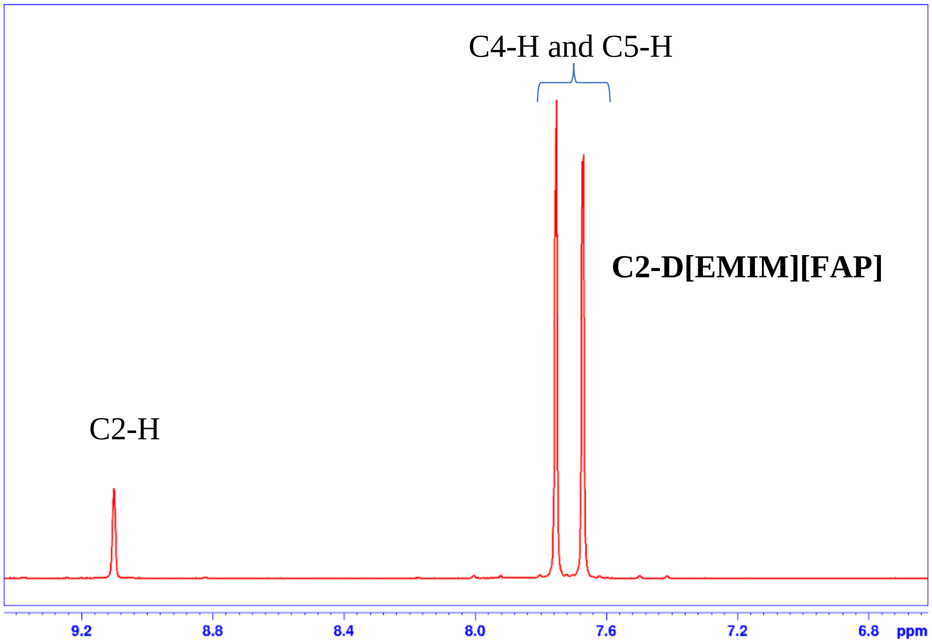

Figure S1. Representative NMR of native [EMIM][FAP] and C2-D labeled [EMIM][FAP] of imidazolium ring protons.

S2. Gaussian fits of 3 % (v/v) DMSO and 10 % (v/v) DMSO in C2-D labeled [EMIM][FAP].

We fit mixtures of DMSO in C2-D labeled [EMIM][FAP] in the C-D region with Gaussian functions using Igor Pro software (Wavemetrics, USA). We fit the most prominent peaks at 2365 cm^{-1} and 2306 cm^{-1} in the 3 % (v/v) DMSO in the ionic liquid and the peak at 2304 cm^{-1} in in the 10 % (v/v) DMSO in the ionic liquid with Gaussian function(s). It should be noted that there are other unresolved peaks particularly in the region between 2348 cm^{-1} and 2320 cm^{-1} in both the mixtures.

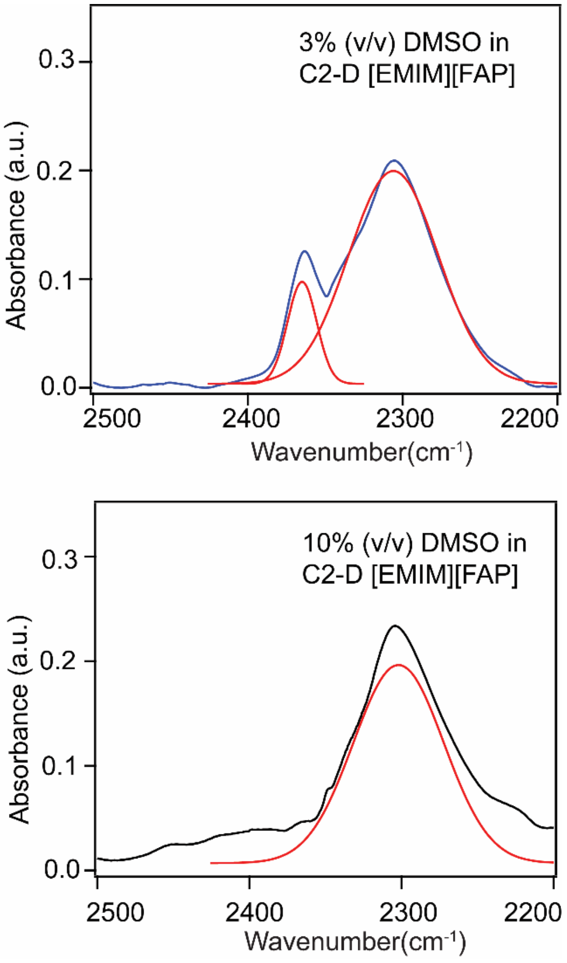

Figure S2. Gaussian fit of mixtures of DMSO with C2-D labeled [EMIM][FAP].

S3. Infrared profile of C-D region of C2-D labeled [EMIM][FAP] on adding DMSO.

Concentration-dependent spectral changes of the C-D region on adding DMSO are shown in Figure S2. The concentrations of DMSO are approximate in the ionic liquid. As observed, on adding DMSO, the C-D peak at 2365 cm^{-1} of C2-D labeled [EMIM][FAP] decreases in intensity and a second broad peak centered at 2306 cm^{-1} with additional unresolved peaks rises in the spectrum. The peak at 2365 cm^{-1} disappears on adding excess DMSO (≥ 10% (v/v)). The second peak slightly red shifts to 2304 cm^{-1} and dominates the C-D region of the ionic liquid solution. However, the changes of intensity of the investigated peak with concentration was not linear.

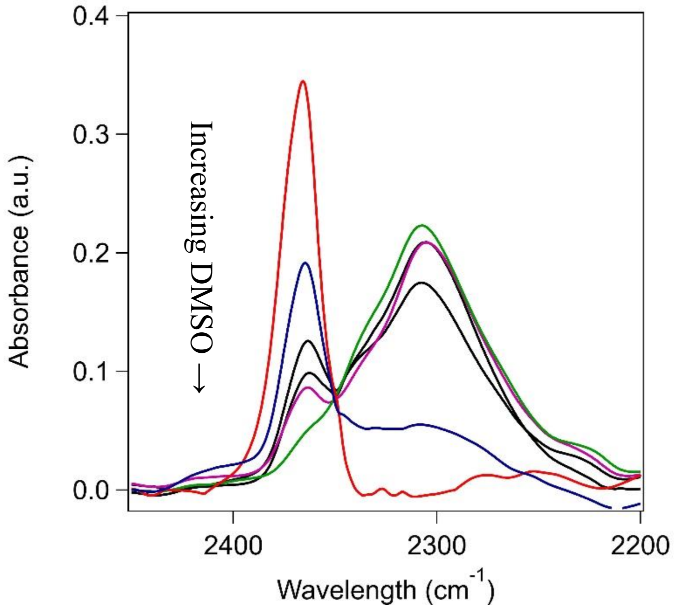

Figure S3. Overlaid infrared signature of the C-D region of pure C2-D labeled [EMIM][FAP] (red), 1% (v/v) of DMSO (blue), 3% (v/v) of DMSO (black), 8% (v/v) of DMSO (pink), and 10 % (v/v) of DMSO (green). Note that the concentrations are approximate.

S4. Changes in C2-D lineshape of C2-D labeled [EMIM][Tf_2N] with DMSO.

DMSO added to [EMIM][Tf_2N] shows similar trend as [EMIM][FAP]. We observe the emergence of a second peak ~2306 cm^{-1} beside the original C-D peak at 2350 cm^{-1} except that is not as well resolved as we observe in C2-D labeled [EMIM][FAP]. We also notice other unresolved peaks at the same region ~2306 cm^{-1} for other ionic liquids on adding DMSO indicating that [EMIM][FAP]-DMSO may be a model system for fluorine-containing anions.

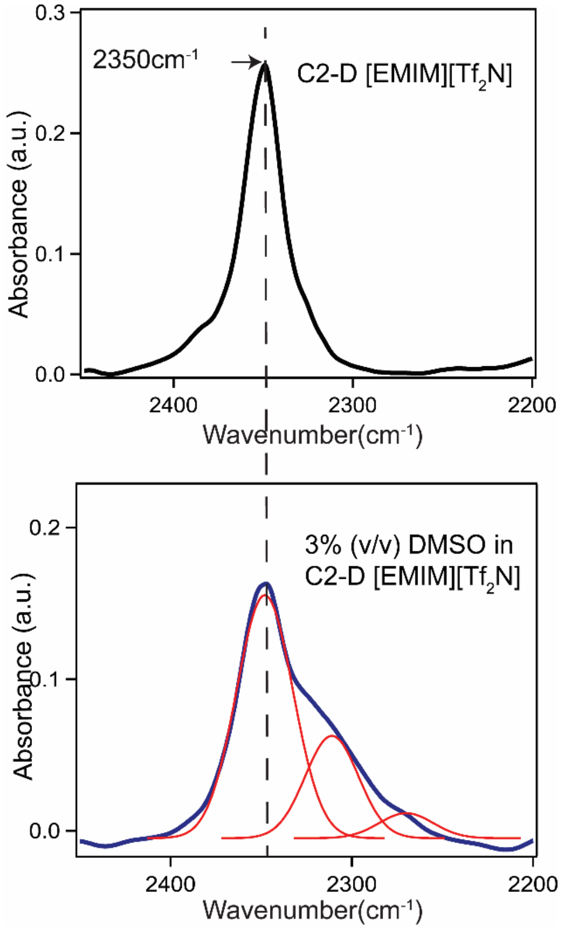

Figure S4. Neat C2-D labeled [EMIM][Tf_2N] (top) and 3 % (v/v) DMSO in C2-D labeled [EMIM] [Tf_2N]. Gaussian fits (red) are inserted for visualization and analysis.

S5. Changes of C-D lineshape of C2-D labeled [EMIM][FAP] with acetone.

Solutions of acetone from 1% (v/v) to 20 % (v/v) in C2-D labeled [EMIM][FAP] do not show the emergence of new peak or red shift of the central C-D peak 2365 cm^{-1} of neat C2-D labeled [EMIM][FAP]. However, there is broadening of the lineshape of the C-D at concentrations greater than 10 % (v/v). A representative figure is shown in Figure S4. It should be noted at concentrations greater than 10 % (v/v), acetone is in excess in C2-D labeled [EMIM][FAP]. We do not see liquid-liquid phase separation under light microscopy and observe characteristic carbonyl stretch of acetone in the infrared spectrum. The result is surprising as our computational studies show that the effect of acetone in [EMIM][FAP] should be similar to DMSO.

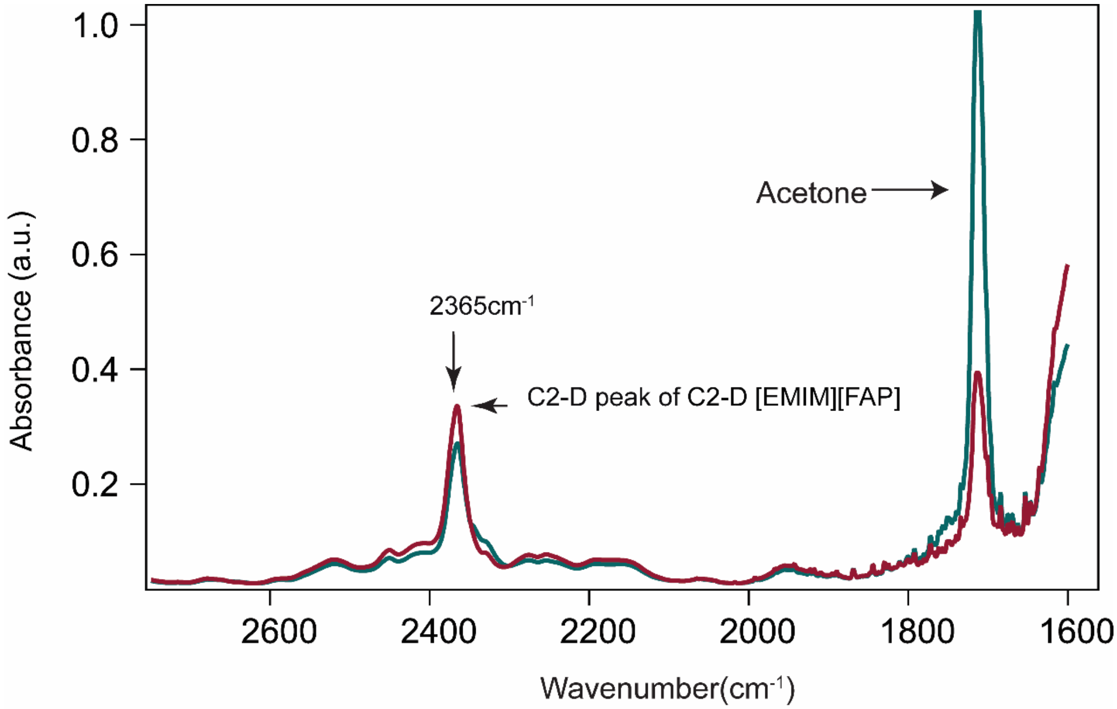

Figure S5. 10% (v/v) (Green) and 2 % (v/v) (Red) of acetone in C2-D labeled [EMIM][FAP].

S6. Computation results.

In order to investigate the geometric preferences for binding of acetone and DMSO to EMIM, we have performed B97M-V/may-cc-pVTZ optimizations of EMIM-acetone and EMIM-DMSO dimers starting from several starting geometries.[1–3] All calculations are run using the CPCM implicit solvation method to mimic the liquid environment. As the particular dielectric constant to be implemented in the CPCM method is unclear, given that the dielectric constant may vary strongly as a function of solute concentration in EMIM/FAP/acetone and EMIM/FAP/DMSO solutions, we have conducted all optimizations (and interaction energy calculations) using six different dielectric constants ranging from 4.9 (corresponding to chloroform) to 24.3 (ethanol). The starting geometries were chosen to represent several different binding motifs that are of particular interest in this study, namely hydrogen bonds involving the C2-H(D), C4-H, and C5-H hydrogens on EMIM, and "stacked" interactions in which acetone/DMSO are located above and below the imidazolium ring (here "above" is denotes a position on the same side as the ethyl tail). Interaction energies for the optimized pairs are computed at the same level of theory (B97M-V/may-cc-pVTZ/CPCM). Harmonic vibrational analyses are carried out, at the same level of theory, only for the most stable hydrogen bonding and stacking complex identified for a given dielectric constant. Examples of starting structures for optimization are given below.

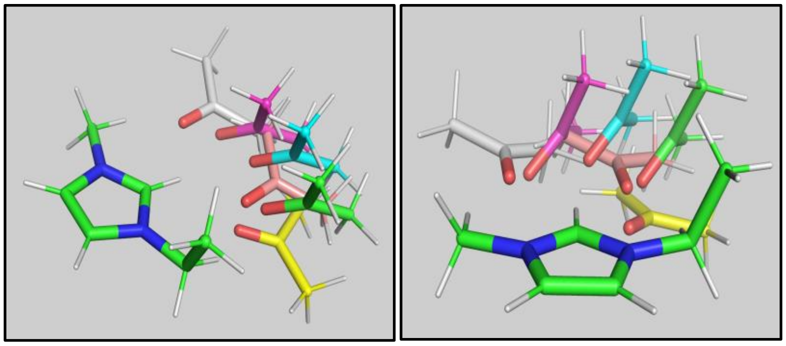

Figure S6. Starting structures for optimization of [EMIM]-acetone structures near the C2-D position. Explanation of labels as indicated by carbon color: H1-C2 (green), H2-C2 (blue), H3-C2 (bright pink), H4-C2 (bright yellow), H5-C2 (pale pink; peach), H6-C2 (white).

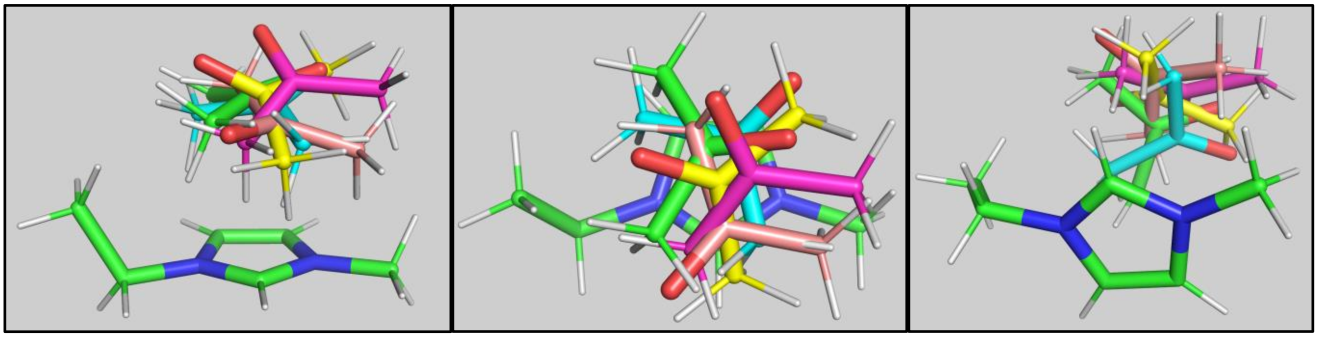

Figure S7. Starting structures for optimization of [EMIM]-acetone structures in "stacked" geometries with acetones located "above" ring. (Here "above" refers to being located in same direction as ethyl tail.) Explanation of labels as indicated by carbon color: H1-C2 (green), H2-C2 (blue), H3-C2 (bright pink), H4-C2 (bright yellow), H5-C2 (pale pink; peach), H6-C2 (white).

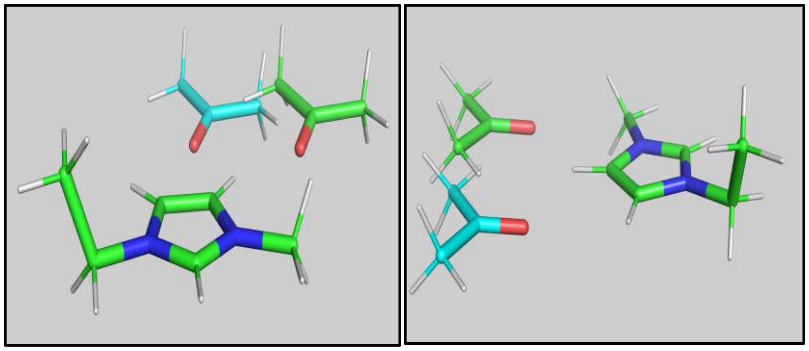

Figure S8. Starting structures for optimization of [EMIM]-acetone near the C4-H position. Explanation of labels as indicated by carbon color: H1-C2 (green), H2-C2 (blue), H3-C2 (bright pink), H4-C2 (bright yellow), H5-C2 (pale pink; peach), H6-C2 (white).

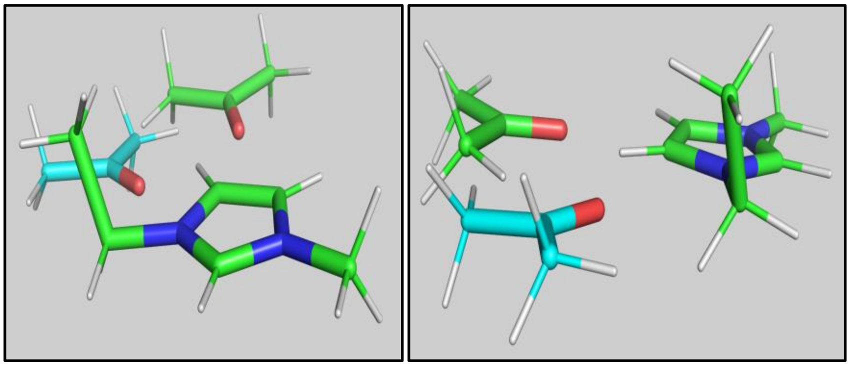

Figure S9. Starting structures for optimization of [EMIM]-acetone near the C5-H position. Explanation of labels as indicated by carbon color: H1-C2 (green), H2-C2 (blue), H3-C2 (bright pink), H4-C2 (bright yellow), H5-C2 (pale pink; peach), H6-C2 (white).

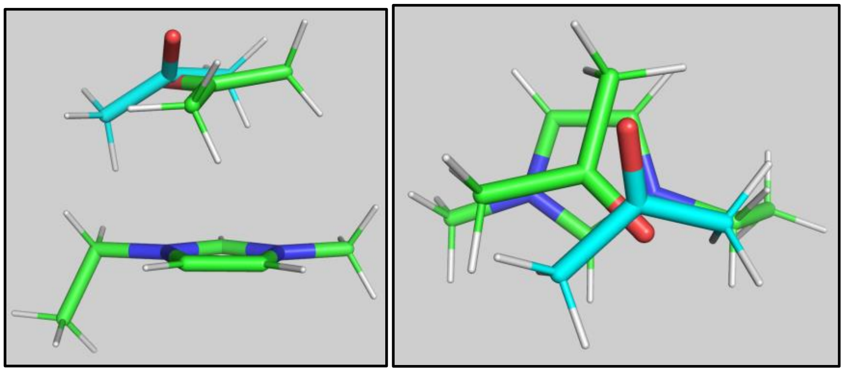

Figure S10. Starting structures for optimization of [EMIM]-acetone structures in "stacked" geometries with acetones located "below" ring. (Here "below" refers to being located in opposite direction as ethyl tail.). Explanation of labels as indicated by carbon color: H1-C2 (green), H2-C2 (blue), H3-C2 (bright pink), H4-C2 (bright yellow), H5-C2 (pale pink; peach), H6-C2 (white).

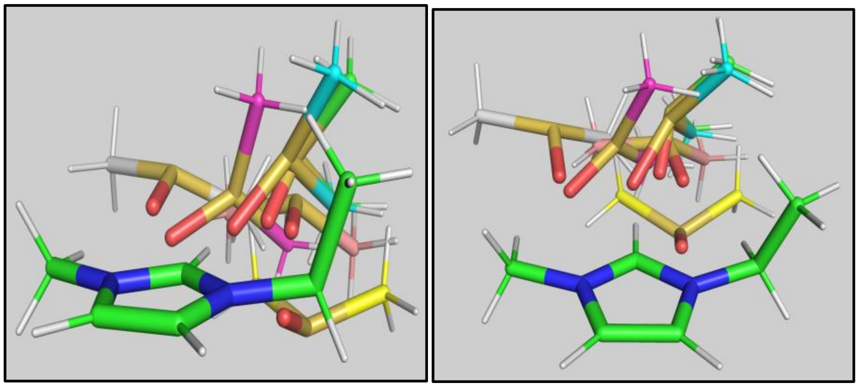

Figure S11. Starting structures for optimization of [EMIM]-DMSO structures near the C2-D position. Explanation of labels as indicated by carbon color: H1-C2 (green), H2-C2 (blue), H3-C2 (bright pink), H4-C2 (bright yellow), H5-C2 (pale pink; peach), H6-C2 (white).

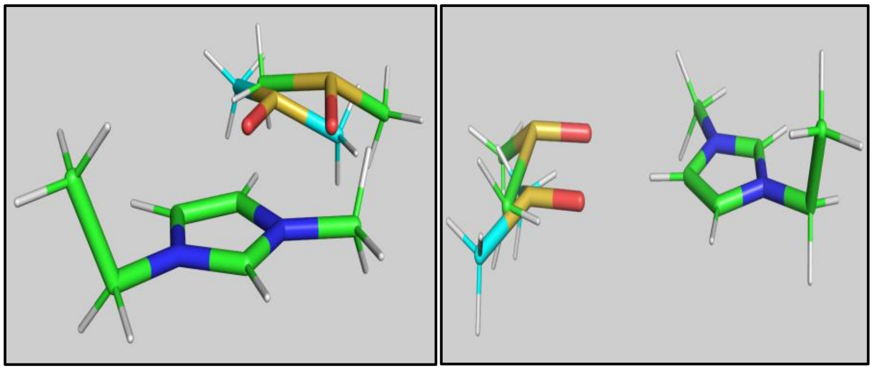

Figure S12. Starting structures for optimization of [EMIM]-DMSO structures near the C4-H position. Explanation of labels as indicated by carbon color: H1-C2 (green), H2-C2 (blue), H3-C2 (bright pink), H4-C2 (bright yellow), H5-C2 (pale pink; peach), H6-C2 (white).

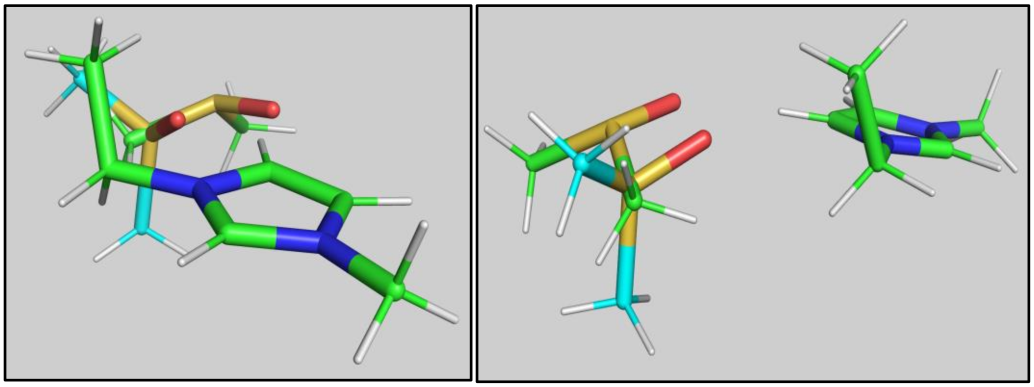

Figure S13. Starting structures for optimization of [EMIM]-DMSO structures near the C5-H position. Explanation of labels as indicated by carbon color: H1-C2 (green), H2-C2 (blue), H3-C2 (bright pink), H4-C2 (bright yellow), H5-C2 (pale pink; peach), H6-C2 (white).

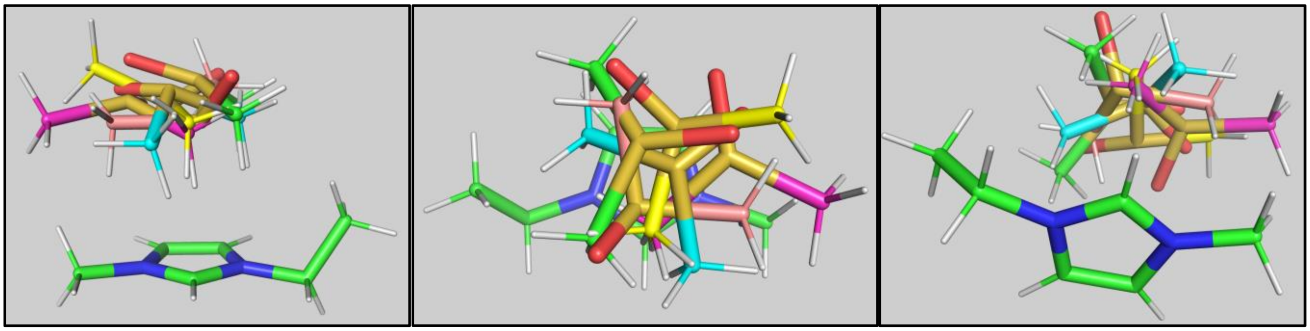

Figure S14. Starting structures for optimization of [EMIM}-DMSO structures in “stacked” geometries with DMSO’s located “above” ring. (Here “above” refers to being located in same direction as ethyl tail.) Explanation of labels as indicated by carbon color: H1-C2 (green), H2-C2 (blue), H3-C2 (bright pink), H4-C2 (bright yellow), H5-C2 (pale pink; peach), H6-C2 (white).

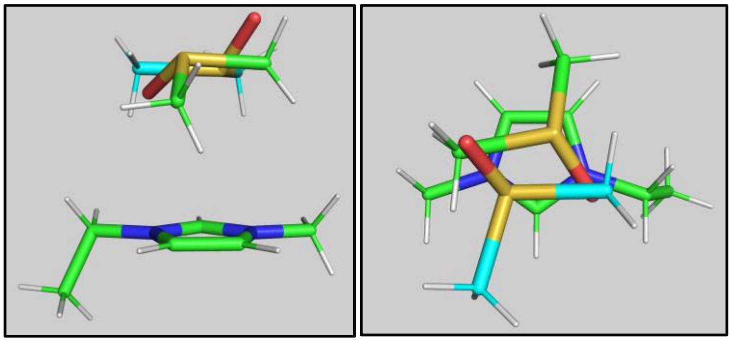

Figure S15. Starting structures for optimization of EMIM-DMSO structures in “stacked” geometries with DMSO’s located “below” ring. (Here “below” refers to being located in opposite direction as ethyl tail.) Explanation of labels as indicated by carbon color: H1-C2 (green), H2-C2 (blue), H3-C2 (bright pink), H4-C2 (bright yellow), H5-C2 (pale pink; peach), H6-C2 (white).

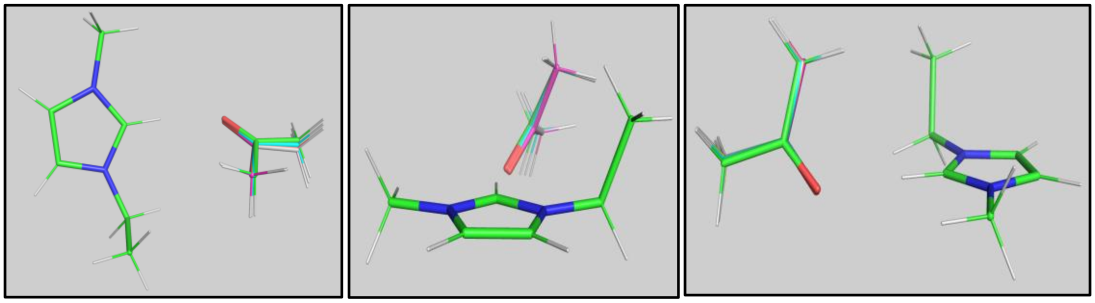

Figure S16. Minimum interaction energy optimized structures for [EMIM]-acetone hydrogen bonding complexes (B97M-V/may-cc-pVTZ) using the CPCM implicit solvation model. Dielectric constants for optimization (corresponding to colors of carbon atoms): green =4.9, blue = 8.0, bright pink = 10.3, bright yellow = 12.5, pale pink (peach) = 17.0, white = 24.3. Note that all of these hydrogen bonds involve the EMIM C2-H(D) deuterium and that dielectric constant has small impact on optimized structures.

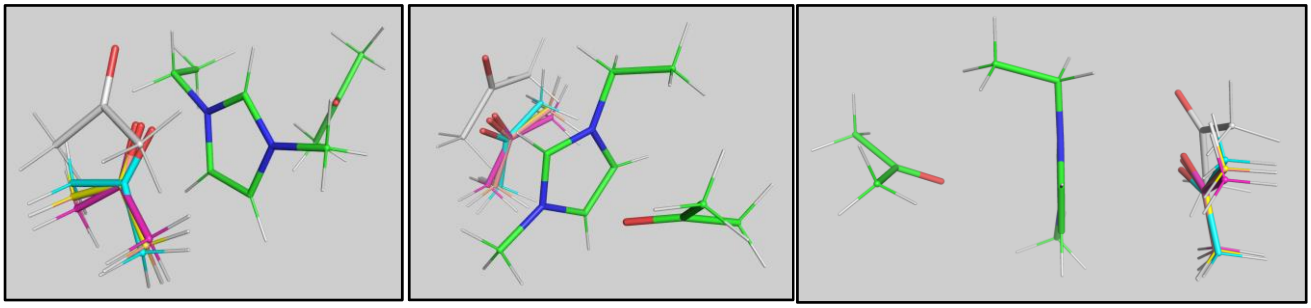

Figure S17. Minimum interaction energy optimized structures for [EMIM]-Acetone “stacked” complexes (B97M-V/may-cc-pVTZ) using the CPCM implicit solvation model. Dielectric constants for optimization (corresponding to colors of carbon atoms): green =4.9, blue = 8.0, bright pink = 10.3, bright yellow = 12.5, pale pink (peach) = 17.0, white = 24.3.

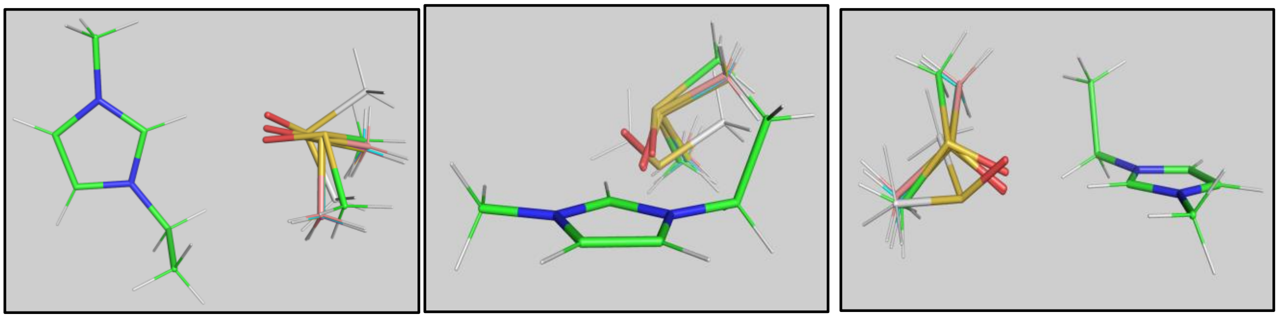

Figure S18. Minimum interaction energy optimized structures for [EMIM]-DMSO hydrogen bonding complexes (B97M-V/may-cc-pVTZ) using the CPCM implicit solvation model. Dielectric constants for optimization (corresponding to colors of carbon atoms): green =4.9, blue = 8.0, bright pink = 10.3, bright yellow = 12.5, pale pink (peach) = 17.0, white = 24.3. Note that all of these hydrogen bonds involve the EMIM C2-H(D) deuterium and optimized structures are very similar for dielectric constants between 8.0 and 17.0.

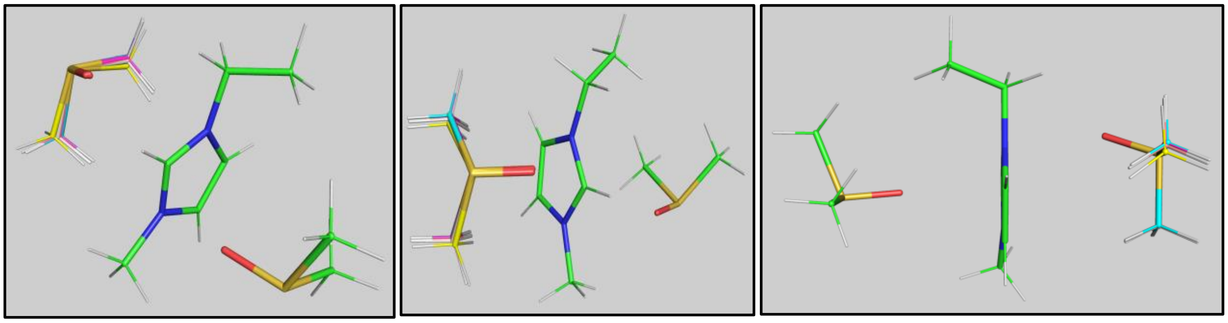

Figure 19. Minimum interaction energy optimized structures for [EMIM]-DMSO “stacked” complexes (B97M-V/may-cc-pVTZ) using the CPCM implicit solvation model. Dielectric constants for optimization (corresponding to colors of carbon atoms): green =4.9, blue = 8.0, bright pink = 10.3, bright yellow = 12.5, pale pink (peach) = 17.0, white = 24.3. Note that optimized structures are very similar for dielectric constants between 8.0 and 24.3.

Table S1. Optimum interaction energies (ΔE(int.)) for EMIM-Acetone and EMIM-DMSO structures with acceptors located near the C2-H(D), C4-H, and C5-H EMIM hydrogens and in "stacking" positions (above; kcal/mol). Maximum C2-D vibrational frequency shifts for C2-D hydrogen bonds and "stacking" positions (below; cm^{-1}).

Dielectric Const.	4.9	8.0	10.3	12.5	17.0	24.3
		**Acetone**		**ΔE(int)**		
**C2-H(D)**	-5.15	-4.35	-4.08	-3.91	-3.69	-3.51
**C4-H**	-4.05	-3.13	-2.82	-2.64	-2.40	-2.21
**C5-H**	-4.63	-3.81	-3.54	-3.38	-3.17	-3.05
**Stacked**	-5.37	-4.69	-4.46	-4.38	-4.26	-4.14
		**DMSO**		**ΔE(int)**		
**C2-H(D)**	-6.76	-5.43	-5.00	-4.74	-4.44	-4.23
**C4-H**	-5.17	-3.97	-3.48	-3.26	-3.00	-2.79
**C5-H**	-5.84	-4.76	-3.95	-3.82	-3.55	-3.37
**Stacked**	-6.08	-4.83	-4.56	-4.38	-4.19	-4.03
		**Acetone**		**Δν**		
**H-bond**	-75.1	-71.7	-70.0	-69.9	-68.3	-66.9
**Stack**	2.8	3.2	3.3	3.4	3.3	3.2
		**DMSO**		**Δν**		
**H-bond**	-98.9	-98.8	-96.1	-95.2	-95.2	-93.8
**Stack**	8.7	12.1	11.8	12.2	11.5	11.4

Table S2. Interaction energies for [EMIM]-acetone structures resulting from optimization (B97M-V/may-cc-pVTZ/CPCM) of various starting structures with all dielectric constants.

[EMIM]-Acetone

Dielectric Const.	4.9	8.0	10.3	12.5	17.0	24.3
			**H-bonds**			
**H-C2**						
h1-c2	-5.151	-4.35	-4.08	-3.91	-3.69	-3.51
h2-c2	-5.150	-4.31	-4.03	-3.86	-3.66	-3.48
h3-c2	-5.099	-4.31	-4.03	-3.86	-3.65	-3.47
h4-c2	-5.083	-4.02	-3.63	-3.40	-3.11	-2.86
h5-c2	-5.132	-4.19	-3.86	-3.66	-3.41	-3.20
h6-c2	-5.145	-4.12	-3.76	-3.55	-3.28	-3.18
**H-C4**						
h1-c4	-4.05	-3.13	-2.82	-2.64	-2.40	-2.21
h2-c4	-3.60	-2.75	-2.44	-2.26	-2.03	-1.83
**H-C5**						
h1-c5	-4.28	-3.58	-3.33	-3.18	-3.00	-2.85
h2-c5	-4.63	-3.81	-3.54	-3.38	-3.17	-3.05
			**Stacked**			
**Top**						
ss1t	-5.37	-4.60	-3.85	-3.81	-3.72	-3.66
ss2t	-5.28	-4.03	-3.89	-3.83	-3.75	-3.70
ss3t	-3.12	-3.04	-3.04	-3.04	-3.05	-3.06
ss4t	-4.23	-3.02	-3.02	-3.03	-3.05	-3.06
ss5t	-4.90	-4.28	-4.07	-3.94	-3.78	-3.64
**Bottom**						
ss1b	-5.20	-4.69	-4.46	-4.38	-4.26	-4.14
ss2b	-4.23	-3.85	-3.73	-3.66	-3.58	-3.51

Minimum interaction energy structures are highlighted in blue, values corresponding to maximum vibrational frequency shifts are highlighted in orange.

Table S3. Interaction energies for [EMIM]-DMSO structures resulting from optimization (B97M-V/may-cc-pVTZ/CPCM) of various starting structures with all dielectric constants considered here.

[EMIM]-DMSO						
Dielectric Const.	4.9	8.0	10.3	12.5	17.0	24.3
**H-bonds**						
**H-C2**						
h1-c2	-6.68	-5.43	-5.00	-4.74	-4.44	-4.16
h2-c2	-6.76	-5.13	-4.72	-4.47	-4.16	-3.90
h3-c2	-6.09	-5.17	-4.85	-4.66	-4.37	-4.17
h4-c2	-6.50	-5.14	-4.67	-4.34	-4.02	-4.23
h5-c2	-6.48	-5.08	-4.63	-4.35	-4.03	-3.78
h6-c2	-6.53	-5.05	-4.59	-4.31	-3.97	-3.74
**H-C4**						
h1-c4	-5.05	-3.88	-3.46	-3.21	-2.90	-2.70
h2-c4	-5.17	-3.97	-3.48	-3.26	-3.00	-2.79
**H-C5**						
h1-c5	-5.84	-4.76	-3.95	-3.82	-3.55	-3.37
h2-c5	-5.72	-4.32	-3.93	-3.70	-3.41	-3.17
**Stacked**						
**Top**						
ss1t	-6.08	-4.34	-4.11	-3.90	-3.83	-3.71
ss2t	-4.70	-4.19	-4.02	-3.88	-3.80	-3.70
ss3t	-6.00	-4.56	-4.29	-4.06	-4.06	-3.92
ss4t	-4.48	-4.18	-3.70	-3.73	-3.76	-3.80
ss5t	-1.67	-2.10	-2.28	-2.39	-2.52	-2.63
**Bottom**						
ss1b	-5.60	-4.83	-4.56	-4.38	-4.19	-4.03
ss2b	-5.51	-4.73	-4.47	-3.13	-4.13	-3.54

Minimum interaction energy structures are highlighted in blue, values corresponding to maximum vibrational frequency shifts are highlighted in orange.

Table S4. C2-D vibrational frequency shifts for all acetone and DMSO structures optimized near the C2-H(D) position of the cation and for optimum (minimum interaction energy) stacked structures.

Dielectric Const.	4.9	8.0	10.3	12.5	17.0	24.3
		**Acetone**		**C2-H(D)**		
h1-c2	-74.5	-71.7	-70.0	-69.9	-68.3	-66.9
h2-c2	-56.0	-67.5	-64.9	-64.8	-63.4	-61.6
h3-c2	-75.1	-70.3	-68.8	-68.4	-66.5	-66.3
h4-c2	-17.8	-15.1	-15.0	-14.8	-14.6	-13.8
h5-c2	-53.9	-55.9	-54.5	-54.1	-53.8	-53.3
h6-c2	-47.7	-51.5	-51.2	-50.9	-50.4	-51.3
		**DMSO**		**C2-H(D)**		
h1-c2	-45.1	-43.3	-41.2	-40.7	-44.5	-48.4
h2-c2	-39.0	-53.0	-52.6	-52.1	-50.8	-52.5
h3-c2	-98.9	-98.8	-96.1	-95.2	-95.2	-93.8
h4-c2	-43.4	-39.4	-39.8	-26.8	-40.8	-75.4
h5-c2	-39.9	-49.2	-48.1	-47.7	-49.3	-47.0
h6-c2	-42.1	-44.9	-45.8	-45.4	-45.3	-48.0
		**Acetone**		**Stacked**		
min	2.8	3.2	3.3	3.4	3.3	3.2
		**DMSO**		**Stacked**		
min	8.7	12.1	11.8	12.2	11.5	11.4

Values corresponding to interaction energy minima are highlighted in blue, maximum C2-D vibrational frequency shifts are highlighted in orange.

References

(1) Neese, F. Software Update: The ORCA Program System—Version 5.0. *Wiley Interdiscip Rev Comput Mol Sci* **2022**, *12* (5), e1606. https://doi.org/10.1002/wcms.1606.

(2) Mardirossian, N.; Head-Gordon, M. Mapping the Genome of Meta-Generalized Gradient Approximation Density Functionals: The Search for B97M-V. *J. Chem. Phys* **2015**, *142* (7), 074111. https://doi.org/10.1063/1.4907719.

(3) Shao, Y.; Molnar, L. F.; Jung, Y.; Kussmann, J.; Ochsenfeld, C.; Brown, S. T.; Gilbert, A. T. B.; Slipchenko, L. V.; Levchenko, S. V.; O'Neill, D. P.; DiStasio, R. A.; Lochan, R. C.; Wang, T.; Beran, G. J. O.; Besley, N. A.; Herbert, J. M.; Yeh Lin, C.; Van Voorhis, T.; Hung Chien, S.; Sodt, A.; Steele, R. P.; Rassolov, V. A.; Maslen, P. E.; Korambath, P. P.; Adamson, R. D.; Austin, B.; Baker, J.; Byrd, E. F. C.; Dachsel, H.; Doerksen, R. J.; Dreuw, A.; Dunietz, B. D.; Dutoi, A. D.; Furlani, T. R.; Gwaltney, S. R.; Heyden, A.; Hirata, S.; Hsu, C. P.; Kedziora, G.; Khalliulin, R. Z.; Klunzinger, P.; Lee, A. M.; Lee, M. S.; Liang, W.; Lotan, I.; Nair, N.; Peters, B.; Proynov, E. I.; Pieniazek, P. A.; Min Rhee, Y.; Ritchie, J.; Rosta, E.; David Sherrill, C.; Simmonett, A. C.; Subotnik, J. E.; Lee Woodcock, H.; Zhang, W.; Bell, A. T.; Chakraborty, A. K.; Chipman, D. M.; Keil, F. J.; Warshel, A.; Hehre, W. J.; Schaefer, H. F.; Kong, J.; Krylov, A. I.; Gill, P. M. W.; Head-Gordon, M. Advances in Methods and Algorithms in a Modern Quantum Chemistry Program Package. *Phys Chem Chem Phys* **2006**, *8* (27), 3172–3191. https://doi.org/10.1039/b517914a.
